# Clinical characteristics and risk factors of non-*Candida* fungaemia

**DOI:** 10.1186/1471-2334-13-247

**Published:** 2013-05-28

**Authors:** Masaki Yamamoto, Shunji Takakura, Gou Hotta, Yasufumi Matsumura, Aki Matsushima, Miki Nagao, Yutaka Ito, Satoshi Ichiyama

**Affiliations:** 1 Department of Clinical Laboratory Medicine, Kyoto University Graduate School of Medicine, 54 Shogoin-Kawaharacho, Sakyo-ku, Kyoto 6068507, Japan; 2 Department of Respiratory Medicine, Kyoto University Graduate School of Medicine, Kyoto, Japan

**Keywords:** Fungaemia, Non-*Candida* yeast, Risk factor, Mortality, Colonisation

## Abstract

**Background:**

The incidence of fungaemia has been increasing worldwide. It is important to distinguish non-*Candida* fungaemia from candidaemia because of their different antifungal susceptibilities. The aims of this study were to investigate the clinical characteristics of non-*Candida* fungaemia and identify the clinical factors that differentiate it from candidaemia.

**Methods:**

We investigated the clinical manifestations and mortality of non-*Candida* fungaemia in Kyoto University Hospital from 2004 to 2009.

**Results:**

There were 110 episodes of fungaemia during the study period. There were 11 renal replacement therapy episodes of fungaemia due to non-*Candida* yeasts (10.0%), including 6 episodes with *Cryptococcus neoformans*, 4 with *Trichosporon asahii*, and 1 with *Kodamaea ohmeri*, in addition to 99 episodes of candidaemia (90.0%). The presence of collagen disease [odds ratio (OR) 9.00; 95% confidence interval (CI) 1.58-51.4; *P* = 0.01] or renal replacement therapy (OR 15.0; 95% CI 3.06-73.4; *P* < 0.01) was significantly more common in non-*Candida* fungaemia patients than in candidaemia patients. Prior colonisation by the species may be a predictor of non-*Candida* fungaemia. Non-*Candida* fungaemia had a higher mortality than candidaemia (54.5% versus 21.2%, *P* = 0.03).

**Conclusions:**

Although *Candida* species frequently cause fungaemia, we should also be aware of non-*Candida* yeasts because of their high mortality, particularly among high-risk patients, such as those with collagen disease and those under renal replacement therapy. Prior colonisation by the causative organisms may be an important predictor of non-*Candida* fungaemia.

## Background

The incidence of hospital-acquired fungaemia caused by yeasts has increased dramatically during the past two decades [1]. This increased incidence has been associated with advances in clinical medicine, including organ transplantation, chemotherapy, antimicrobial agents, parenteral nutrition, and medical devices, all of which improve patient survival but increase the risk of infection [2]. *Candida* species are the leading cause of yeast fungaemia. However, as the fungaemia patient population has changed, several types of rare yeasts have become recognised pathogens, particularly in nosocomial settings, and have increased in clinical importance [1]. These yeasts, which include *Trichosporon* species, *Cryptococcus* species, *Rhodotorula* species, *Malassezia* species, and *Blastoschizomyces capitatus*, have been associated with life-threatening infections in immunocompromised patients [3-6].

The mortality rate among patients with fungaemia is high, ranging from 50% to 80% [1,7-10]. The variable susceptibility profiles to antifungal agents are one of the major reasons for the poor prognosis of these yeast infections. *Candida* species are usually susceptible to standard antifungal agents. However, the treatment of non-*Candida* yeasts is challenging because of their rarity and the prevalence of in vitro resistance to standard antifungal agents [11]. For example, *Cryptococcus* species are resistant to echinocandins, and *Trichosporon* species are characterised by resistance to amphotericin and echinocandins. For these reasons, early distinction between non-*Candida* species and *Candida* species is important.

Although the importance of non-*Candida* yeasts is recognised, little is known about the epidemiology and risk factors associated with non-*Candida* fungaemia. To evaluate the clinical characteristics of non-*Candida* fungaemia and determine the risk factors associated with non-*Candida* fungaemia, we conducted a retrospective cohort study of yeast fungaemia detected in a tertiary-care university hospital over a 6-year period.

## Methods

### Study population

From 2004 to 2009, all patients with non-*Candida* fungaemia were identified through the records of the clinical microbiological laboratory at Kyoto University Hospital, a tertiary-care, 1182-bed university hospital in Japan. The medical records of all patients with yeast fungaemia were reviewed. Patients were included if they had evidence of sepsis with at least one positive blood culture containing yeasts. Only one episode per patient was included in this study. The Institutional Review Board of Kyoto University Hospital approved this study protocol.

### Clinical characteristics and risk factor analysis

Demographic data, the potential risk factors for developing yeast fungaemia presenting within 30 days prior to the diagnosis of fungaemia, and outcomes were retrieved from the medical records. The following data were recorded: age, gender, hospital-acquired infection (HAI), days of hospitalisation prior to the onset of fungaemia, admission ward at the onset of fungaemia, co-morbidities (solid malignancy, diabetes mellitus, gastrointestinal/hepatobiliary disease, chronic kidney disease, haematologic malignancy, cardiovascular disease, and collagen disease), underlying conditions [prior intensive care unit admission, neutropenia, colonisation by the causative yeast, polymicrobial bacteraemia, assisted ventilation, renal replacement therapy (haemodialysis and continuous haemodiafiltration), indwelling Foley catheter, central venous catheterisation, parenteral nutrition, enteral nutrition, corticosteroid therapy, chemotherapy, other immunosuppressant therapy, prior antibiotic therapy, prior antifungal therapy, prior surgical procedures (abdominal or other), the severity of illness, and 30-day mortality.

### Species identification and antifungal susceptibility testing

Blood cultures were performed using the BacT/ALERT automated culture system (bioMerieux, Marcy-l’Etoile, France). Species identification was performed with standard laboratory procedures, including morphological identification and the API 20C AUX system (bioMerieux). The minimum inhibitory concentrations (MICs) of each antifungal drug were determined using the reference broth microdilution method and interpreted according to the M27-A2 guidelines of the Clinical and Laboratory Standards Institute [12].

### Definitions

HAI was defined as an infection acquired at least 48 hours after hospitalisation that was not clinically apparent at the time of hospitalisation. Neutropenia was defined as an absolute neutrophil count of less than 500 cells/μL. Polymicrobial bacteraemia was defined as the isolation of other bacteria from the blood within 24 hours of the initial positive fungal culture. Fungal colonisation was defined as a positive culture of causative yeasts from any bodily site other than blood before the onset of fungaemia, with no clinical sign or symptom of infection at that site. Corticosteroid therapy was defined as administration of at least 20 mg of a prednisone equivalent for at least 1 week (this value was adjusted according to age for paediatric patients). The severity of illness was estimated using the sequential organ failure assessment (SOFA) score on the day of fungaemia onset.

### Statistics

Statistical analyses were performed using SPSS version 18.0 (SPSS Inc., Chicago, IL, USA). Fisher’s exact or Pearson’s chi-square test was used as appropriate to compare categorical variables. The Mann–Whitney *U* test was used to test for the statistical significance of continuous variables. Multivariate logistic regression analysis was used for risk factor analysis for non-*Candida* fungaemia and outcome analysis. A forward selection method was used with the entry criterion of a *P* value <0.1 for clinical factors associated with risk factors of non-*Candida* fungaemia, and factors with a *P* value <0.05 were retained in the final model. A *P* value of <0.05 was considered statistically significant.

## Results

During the study period, 102 yeasts were isolated from 110 patients with fungaemia. No patient had evidence of human immunodeficiency virus infection. Eleven of 110 episodes (10.0%) were non-*Candida* fungaemia. Of the 110 episodes of yeast fungaemia, 112 yeast isolates were identified; 2 of 110 episodes showed coinfection with 2 *Candida* isolates. Among these yeast isolates, 11 (9.8%) were non-*Candida* yeasts, and 101 (90.2%) were *Candida* species. Among the 11 non-*Candida* isolates, *Cryptococcus neoformans* (6 isolates, 5.4%), *Trichosporon asahii* (4 isolates, 3.6%), and *Kodamaea ohmeri* (1 isolate, 0.9%) were identified. Among the 101 *Candida* isolates, the most common was *Candida albicans* (46 isolates, 41.1%), followed by *Candida parapsilosis* (22 isolates, 19.6%), *Candida glabrata* (15 isolates, 13.4%), *Candida tropicalis* (9 isolates, 8.0%), and *Candida guilliermondii* (5 isolates, 4.5%). *Candida krusei*, *Candida lusitaniae*, and *Candida famata* were isolated in 1 episode each. Another *Candida* isolate could not be identified to the species level.

All 6 *C. neoformans* isolates showed low MIC values for amphotericin (0.125-0.25 mg/L) and fluconazole (1–8 mg/L) and high MIC values for micafungin (≥32 mg/L). In the 4 isolates of *T. asahii*, the MIC values for fluconazole ranged from 4 mg/L to 8 mg/L, and the MIC values for micafungin were ≥16 mg/L. *K. ohmeri* showed low MIC values for amphotericin (0.25 mg/L), fluconazole (≤0.125 mg/L), and micafungin (0.25 mg/L). Nine of the 112 isolates of *Candida* species were resistant to fluconazole (8.0%).

The medical records were available for all 110 patients with fungaemia. Table 1 shows the clinical characteristics of patients with non-*Candida* fungaemia. Immunosuppressants were used in 7 cases (63.6%). Five patients (45.5%) were under renal replacement therapy. Colonisation by causative organisms was identified in 8 patients (72.7%), and urine was the major site of colonisation. Initial treatment with antifungal agents at a high MIC was found in 3 of 4 episodes of *T. asahii* fungaemia. Two episodes were treated with micafungin, and 1 was treated with fluconazole. In cryptococcaemia, a lack of antifungal therapy was identified in 2 cases because these 2 patients died before starting antifungal therapy, and others were treated with antifungal agents at a low MIC. A treatment delay of over 2 days or lack of antifungal therapy was noted for 8 patients (72.7%).

**Table 1:** **Clinical characteristics of non-*Candida *fungaemia infection**

	***C. neoformans*(n = 6)**	***T. asahii*(n = 4)**	***K. ohmeri*(n = 1)**
Co-morbidities^a^	6 (100%)	4 (100%)	1 (100%)
Immunosuppressant therapy	4(66.7%)	2 (50.0%)	1 (100%)
Renal replacement therapy	2 (33.3%)	2 (50.0%)	1 (100%)
Colonisation by causative yeasts	4 (66.7%)	4 (100%)	1 (100%)
SOFA score, median (range)	9 (1–18)	8 (1–17)	20 (−)
In vitro-active antifungal agent	4 (66.7%)	1 (25.0%)	1 (100%)
30-day mortality	3 (50.0%)	3 (75.0%)	0 (0%)

*SOFA*: sequential organ failure assessment.

^a^ Co-morbidities of *C. neoformans* infection include acute respiratory distress syndrome, hepatitis C virus infection, post-living donor liver transplantation (LDLT), systemic lupus erythaematosus, and miliary tuberculosis. Co-morbidities of *T. asahii* infection include POEMS syndrome, liver cirrhosis, Bechet’s disease, and acute myeloid leukaemia. Co-morbidities of *K. ohmeri* infection include alcoholic liver cirrhosis and post-LDLT.

The demographic characteristics and clinical manifestations of the patients with fungaemia are shown in Table 2. The median age of non-*Candida* fungaemia patients was 66 years (interquartile range 60–71 years), and 45.5% were female. There was no significant difference in age (*P* = 0.80), gender (*P* = 0.57), frequency of HAI (*P* = 0.65), or days of hospitalisation prior to fungaemia onset (*P* = 0.62) between the non-*Candida* fungaemia and candidaemia groups. Non-*Candida* fungaemia had a higher mortality rate than candidaemia (54.5% versus 21.2%, *P* = 0.03).

**Table 2:** **Demographic characteristics and treatment outcomes of patients with fungaemia (*N *= 110)**

**Characteristics**	**Type of fungaemia,*N*(%)**			***P*value**
	**Non-*Candida*fungaemia**	**Candidaemia**	**Overall**	
	11 (10.0)	99 (90.0)	110 (100)	
Age, years ^a^	66 (60–71)	65 (49–74)	65 (54–73)	0.80
Female gender	5 (45.5)	54 (54.5)	59 (53.6)	0.57
Hospital-acquired infection	11 (100)	95 (96.0)	106 (96.4)	0.65
Days of prior hospitalisation ^a, b^	47 (11–66)	30 (12–60)	30 (12–60)	0.62
Admission ward at fungaemia onset				0.71
Surgical ward	3 (27.3)	40 (40.4)	43 (39.1)	
Medical ward	6 (54.5)	41 (41.4)	47 (42.7)	
ICU	2 (18.2)	14 (14.1)	16 (14.5)	
Co-morbidities	11 (100)	92 (92.9)	103 (93.6)	0.47
Solid malignancy	0 (0)	45 (45.5)	45 (40.9)	< 0.01*
Diabetes mellitus	5 (45.5)	25 (25.3)	30 (27.3)	0.14
Gastrointestinal/hepatobiliary disease	6 (54.5)	60 (60.6)	66 (60.0)	0.47
Chronic kidney disease	6 (54.5)	18 (18.2)	24 (21.8)	0.01*
Haematologic malignancy	1 (9.1)	14 (14.1)	15 (13.6)	0.54
Cardiovascular disease	3 (27.3)	17 (17.2)	20 (18.2)	0.32
Collagen disease	3 (27.3)	9 (9.1)	12 (10.9)	0.09*
Underlying conditions				
Prior ICU admission	5 (45.5)	31 (31.3)	36 (32.7)	0.27
Neutropaenia	1 (9.1)	7 (7.1)	8 (7.3)	0.58
Prior bacteraemia	7 (63.6)	32 (32.3)	39 (35.5)	0.04*
Colonisation by causative yeasts	8 (72.7)	41 (41.4)	49 (44.5)	0.05*
Polymicrobial bacteraemia	5 (45.5)	23 (23.2)	28 (25.5)	0.11
Assisted ventilation	2 (18.2)	17 (17.2)	19 (17.3)	0.60
Renal replacement therapy	5 (45.5)	9 (9.1)	14 (12.7)	< 0.01*
Indwelling Foley catheter	7 (63.6)	42 (42.4)	49 (44.5)	0.15
Central venous catheterisation	8 (72.7)	82 (82.8)	90 (8.18)	0.32
Parenteral nutrition	8 (72.7)	80 (80.8)	88 (80.0)	0.38
Enteral nutrition	9 (81.8)	56 (56.6)	65 (59.1)	0.09*
Corticosteroid therapy	7 (63.6)	31 (31.3)	38 (34.5)	0.04*
Chemotherapy	0 (0)	27 (27.3)	27 (24.5)	0.04*
Other immunosuppressant therapy	3 (27.3)	8 (8.1)	11 (10.0)	0.08*
Prior antibiotic therapy	10 (90.9)	89 (89.9)	99 (90.0)	0.70
Prior antifungal therapy	4 (36.4)	18 (18.2)	22 (20.0)	0.15
Prior surgical procedures	2 (18.2)	28 (28.3)	30 (27.3)	0.38
Abdominal surgery	1 (9.1)	19 (19.2)	20 (18.2)	0.37
Other surgery	1 (9.1)	10 (10.1)	11 (10.0)	0.70
SOFA score ^a^	11 (3–17)	3 (1–9)	3.5 (1.8-10)	0.02*
Death within 30 days	6 (54.5)	21 (21.2)	27 (24.5)	0.03*

*ICU*: intensive care unit; *SOFA*: sequential organ failure assessment.

Data are presented as median values (interquartile range) for continuous variables and numbers of cases (%) for categorical variables.

*P* values are marked in bold if <0.05.

^a^ Variables categorised as an ordinal scale in 1-point increments: OR > 1 is the increase in likelihood of the outcome with a 1-point increase in the factor.

^b^ Days of hospitalisation prior to fungaemia onset.

* *P* < 0.10, variable was included in the multivariate analysis of outcomes from non-*Candida* fungaemia versus candidaemia.

The risk factors in patients with yeast fungaemia are shown in Table 3. In the univariate analysis, factors significantly associated with non-*Candida* fungaemia were chronic kidney disease [odds ratio (OR) 5.40; 95% confidence interval (CI) 1.48-19.7; *P* = 0.01], prior bacteraemia (OR 3.66; 95% CI 1.00-13.4; *P* = 0.04), renal replacement therapy (OR 8.33; 95% CI 2.12-32.8; *P* < 0.01), corticosteroid therapy (OR 3.84; 95% CI 1.05-14.1; *P* = 0.04), and higher SOFA score (OR 1.13; 95% CI 1.03-1.26; *P* = 0.02). In the multivariate analysis, independent risk factors associated with non-*Candida* fungaemia included collagen disease (OR 9.00; 95% CI 1.58-51.4; *P* = 0.01) and renal replacement therapy (OR 15.0; 95% CI 3.06-73.4; *P* < 0.01).

**Table 3:** **Risk factors predicting non-*Candida *fungaemia**

**Variables**	**Univariate analysis**		**Multivariate analysis**	
	**Crude OR (95% CI)**	***P*value**	**Adjusted OR (95% CI)**	***P*value**
Co-morbidities				
Solid malignancy	0.55 (0.46-0.65)	**< 0.01**	-	-
Chronic kidney disease	5.40 (1.48-19.7)	**0.01**	-	-
Collagen disease	3.75 (0.84-16.7)	0.09	9.00 (1.58-51.4)	**0.01**
Underlying conditions				
Prior bacteraemia	3.66 (1.00-13.4)	**0.04**		
Colonisation by causative yeasts	3.77 (0.94-15.1)	0.05	-	-
Renal replacement therapy	8.33 (2.12-32.8)	**< 0.01**	15.0 (3.06-73.4)	**< 0.01**
Corticosteroid therapy	3.84 (1.05-14.1)	**0.04**	-	-
Chemotherapy	0.73 (0.65-0.82)	**0.04**	-	-
Other immunosuppressant therapy	4.27 (0.94-19.3)	0.08		
SOFA score ^a^	1.13 (1.03-1.26)	**0.02**	-	-

*OR*: odds ratio; *SOFA*: sequential organ failure assessment.

*P* values are marked in bold if < 0.05.

^a^ Variables categorised as an ordinal scale in 1-point increments: OR > 1 is the increase in likelihood of the outcome with a 1-point increase in the factor.

*P* < 0.10, variable was included in the multivariate analysis of outcomes from non-*Candida* fungaemia versus candidaemia.

## Discussion

Non-*Candida* yeasts, which are clinically less common than *Candida* species, have been associated with life-threatening infections in immunocompromised individuals. Although the importance of these opportunistic yeasts is recognised, little is known about their epidemiology [11]. In this study, similar to previous studies, fungaemia due to non-*Candida* yeasts was less common than candidaemia, but it still accounted for a significant proportion of all fungaemia episodes (10.0%). This incidence was similar to the rate of fluconazole-resistant *Candida* among candidaemia in our hospital (8.0%). The incidence of non-*Candida* fungaemia was slightly higher than in previous reports, which have reported rates of 3-6% [2,3]. One possible reason for this finding is that we included fungaemia caused by *C. neoformans* in this study because of its different susceptibility profile to antifungal agents compared to *Candida* species. *Cryptococcus* species are resistant to echinocandins [13]. However, micafungin, an echinocandin, is commonly used for initial empirical therapy for yeast fungaemia in our institute.

Among patients with non-*Candida* fungaemia, one or more co-morbidities were identified in all patients, but none had a solid malignancy. Several factors, including chronic kidney disease, prior bacteraemia, renal replacement therapy, corticosteroid therapy, higher SOFA score, and 30-day mortality, were significantly over-represented in non-*Candida* fungaemia. However, independent risk factors associated with non-*Candida* fungaemia only included collagen disease and renal replacement therapy. Other significant risk factors for non-*Candida* fungaemia in the univariate analysis might have confounded these associations. Although colonisation by *Candida* species at multiple sites in the body is commonly recognised as a major risk factor for invasive candidiasis in critically ill patients, *Candida* colonisation occurs primarily in immunocompromised patients [14]. In this study, colonisation by causative organisms prior to fungaemia onset was noted in 72.7% of non-*Candida* fungaemia patients. This finding is intriguing and may be an important clue for predicting the occurrence of non-*Candida* fungaemia.

Non-*Candida* fungaemia was significantly associated with 30-day mortality in the univariate analysis but was not an independent predictor in the multivariate analysis. Although this factor could be a confounding variable, the mortality of non-*Candida* fungaemia was higher than that of candidaemia (54.5% versus 21.2%). This high mortality rate is consistent with previous reports [2,3]. However, the mortality of candidaemia (21.2%) was lower compared to those of previous reports (50-80%) [1,7-10]. The difference in the severity of illness might explain this discrepancy. Anunnatsiri *et al.* reported that patients with non-*Candida* fungaemia and patients with candidaemia had similar illness severities [2]. In our study, patients with candidaemia had a lower SOFA score than patients with non-*Candida* fungaemia (*P* = 0.02), although this difference was not significant in the multivariate analysis. Another explanation for this discrepancy is that the clinical intervention by infectious disease physicians contributed to the low mortality of candidaemia in our hospital [15].

Inappropriate therapy is a significant predictor of mortality in fungaemia [7,10,16-19]. Antifungal resistance is common among non-*Candida* yeasts; thus, treatment options are limited, and the appropriate therapy can be left untried [11]. Initial therapy with antifungals at a high MIC was primarily found in *T. asahii* fungaemia (75%). However, the rate of appropriate therapy was similarly low between non-*Candida* fungaemia and candidaemia when appropriate therapy was defined as treatment with agents at a low MIC within 24 hours (data not shown). These findings suggest that other factors (e.g., background and comorbidities) might contribute to the high mortality of non-*Candida* fungaemia.

Our results reveal important insights into the epidemiology of non-*Candida* fungaemia. However, this study had several limitations. The most important limitation was the small sample size of non-*Candida* fungaemia patients because of low incidence. In addition, other rare opportunistic yeasts (e.g., *Rhodotorula* species, *Saccharomyces* species, and *Malassezia* species) were not detected in this study. Second, due to the retrospective nature of the study, we did not identify the focus or cause of infection in some cases; therefore, we could not address the application of appropriate therapy in all non-*Candida* fungaemia patients. Furthermore, we could not carry out molecular species identification. Finally, this study only evaluated the crude mortality of patients with fungaemia. The development of national databases and well-defined multicentre studies are needed to resolve these limitations.

## Conclusions

In conclusion, although non-*Candida* fungaemia is a rare cause of yeast fungaemia, it should receive more attention because of its high mortality, particularly among high-risk patients, such as those with collagen disease and those under renal replacement therapy. Prior colonisation by causative organisms may be an important predictor of non-*Candida* fungaemia.

## Competing interests

The authors declare that they have no competing interests.

## Authors’ contributions

MY conceived the study, participated in its design, reviewed the medical records, performed the statistical analysis, and drafted the manuscript. ST and SI participated in the design of the study, coordination, and manuscript preparation. GH, YM, AM, and MN participated in manuscript preparation. All authors read and approved the final manuscript.

## Pre-publication history

The pre-publication history for this paper can be accessed here:

http://www.biomedcentral.com/1471-2334/13/247/prepub
